# Chicken Interferon-Inducible Transmembrane Protein 3 Restricts Influenza Viruses and Lyssaviruses *In Vitro*

**DOI:** 10.1128/JVI.01443-13

**Published:** 2013-12

**Authors:** S. E. Smith, M. S. Gibson, R. S. Wash, F. Ferrara, E. Wright, N. Temperton, P. Kellam, M. Fife

**Affiliations:** Wellcome Trust Sanger Institute, Wellcome Trust Genome Campus, Hinxton, United Kingdoma; The Pirbright Institute, Compton Laboratory, Compton, Berkshire, United Kingdomb; Viral Pseudotype Unit, School of Pharmacy, University of Kent, Chatham Maritime, Kent, United Kingdomc; Viral Pseudotype Unit (Fitzrovia), School of Life Sciences, University of Westminster, London, United Kingdomd; MRC/UCL Centre for Medical Molecular Virology, Division of Infection and Immunity, University College London, London, United Kingdome

## Abstract

Interferon-inducible transmembrane protein 3 (IFITM3) is an effector protein of the innate immune system. It confers potent, cell-intrinsic resistance to infection by diverse enveloped viruses both *in vitro* and *in vivo*, including influenza viruses, West Nile virus, and dengue virus. IFITM3 prevents cytosolic entry of these viruses by blocking complete virus envelope fusion with cell endosome membranes. Although the IFITM locus, which includes *IFITM1*, -*2*, -*3*, and -*5*, is present in mammalian species, this locus has not been unambiguously identified or functionally characterized in avian species. Here, we show that the IFITM locus exists in chickens and is syntenic with the IFITM locus in mammals. The chicken IFITM3 protein restricts cell infection by influenza A viruses and lyssaviruses to a similar level as its human orthologue. Furthermore, we show that chicken IFITM3 is functional in chicken cells and that knockdown of constitutive expression in chicken fibroblasts results in enhanced infection by influenza A virus. Chicken *IFITM2* and -*3* are constitutively expressed in all tissues examined, whereas *IFITM1* is only expressed in the bursa of Fabricius, gastrointestinal tract, cecal tonsil, and trachea. Despite being highly divergent at the amino acid level, IFITM3 proteins of birds and mammals can restrict replication of viruses that are able to infect different host species, suggesting IFITM proteins may provide a crucial barrier for zoonotic infections.

## INTRODUCTION

Type I and II interferons (IFNs) are critical for the development of the cell intrinsic antiviral state and achieve this by inducing the expression of genes collectively named IFN-stimulated genes (ISGs). Expression of the interferon-inducible transmembrane (*IFITM*) genes (new members of the ISG family) restricts the replication of several highly pathogenic human viruses, including severe acute respiratory syndrome (SARS) coronavirus, filoviruses (Marburg virus and Ebola virus), influenza A viruses (IAVs), and flaviviruses (dengue virus) (1, 2). Although restriction of HIV-1 infection has also been reported in some studies (3, 4), others have failed to demonstrate such activity (1). IFITM proteins are small, with an average size of 130 amino acids, and share a topology (5) defined by a conserved CD225 domain (6). This domain consists of two intramembrane (IM) regions and a conserved intracellular loop (CIL).

As their names suggest, IFITM proteins are upregulated by type I and II IFNs; however, some cell and tissue types express constitutive levels of one or more of these proteins (7). In humans, *IFITM1*, -*2*, and -*3* are expressed in a wide range of tissues, while *IFITM5* expression is limited to osteoblasts. Mice have orthologues for *IFITM1*, -*2*, -*3*, and -*5* and additional IFITM genes, *Ifitm6* and *Ifitm7* (8). Genome analysis of chickens has predicted the existence of two IFITM genes, orthologous to human *IFITM10* (*huIFITM10*) and *huIFITM5* (9). However, such *in silico* analysis is often confounded by inappropriate identification of pseudogenes and incorrect assignment of orthologues, due to an incomplete knowledge of IFITM gene duplication and evolutionary history of this locus during speciation. Under such circumstances, careful genome analysis of syntenic regions and functional characterization of genes are required to attempt to unambiguously define orthologous genes.

IFITM proteins are the only mediators of innate immunity known to inhibit viral infection by blocking cytoplasmic entry and replication of diverse enveloped viruses (10). IFITM-mediated viral restriction occurs at entry sites of susceptible viruses, in the late endosomal and lysosomal compartments, where the proteins are predicted to adopt an intramembrane structure. The N and C termini of the proteins are predicted to be cytoplasmic, the two intramembrane domains are buried within the cytoplasmic facing lipid bilayer, and the CD225 domain is thought to be facing the cytoplasm (11). IFITM proteins inhibit formation of a fusion pore between the virus and endosomal membranes following acidic activation of virus envelope fusion proteins. Recently, the ability of IFITM proteins to alter cellular membrane fluidity was demonstrated, leading to the arrest of fusion pore formation at the stage of hemimembrane fusion (12). Furthermore, it was recently found that IFITM3 interacts with vesicle-membrane-protein-associated protein A (VAPA) and prevents its association with oxysterol-binding protein (OSBP) (13), which disturbs intracellular cholesterol homeostasis and thus causes inhibition of viral fusion in the late endosome, by an unknown mechanism.

The high constitutive levels of IFITM proteins observed in many tissues potentially provide a first line of defense against virus infection. The induction of type I IFNs further promotes IFITM expression, increasing their protective effect on surrounding uninfected cells. Depletion of *Ifitm3* in mouse cells results in a loss of 40% to 70% of IFN′s protective effect against endosomal entering viruses (1). A similar attenuation is also observed from cells derived from *IfitmDel* mice, lacking *Ifitm1*, -*2*, -*3*, -*5*, and *6*, suggesting IFITM3 accounts for most of the antiviral activity of this locus for the viruses investigated (14). Importantly, mice homozygous for *Ifitm3* deletion suffer fulminant viral pneumonia when challenged with low-pathogenicity IAV (14, 15). Direct clinical relevance of IFITM3's involvement in restricting human IAV infection has recently been shown in individuals hospitalized with seasonal or pandemic influenza H1N1/09 viruses (15), where a statistically significant number of hospitalized patients show overrepresentation of a minor *C* allele in *IFITM3* (*rs12252-C*) that correlates with a decrease in the ability of *IFITM3* to restrict influenza virus infection *in vitro*. Importantly, the significance of the association of the *rs12252-C* allele with severe influenza infection was recently replicated in a Chinese cohort of patients (16). Together, these data reveal that the action of IFITM3 profoundly alters the course of influenza virus infection in mammals and that allelic variation in IFITM3 alters host susceptibility to severe influenza virus infection. Although IFITMs have been well characterized in humans and mice, little compelling functional data exists for this ISG family in other species.

Avian IAVs represent a continuing threat to human populations both as a source for direct human infection and as a reservoir for IAV genetic variation. These reservoirs provide the conditions for the generation of reassorted IAVs with altered host ranges and pandemic potential (17). Furthermore, endemic and emerging avian viral pathogens create major challenges to the poultry industry through loss of productivity and mortality. Similarly, lyssaviruses, particularly rabies virus (RABV), pose a substantial public health threat, with half of the world's population living in areas of endemicity (18), although reports of avian lyssavirus infections are rare. The clinical presentations of an infection are identical for all lyssavirus species; however, while current vaccines and postexposure prophylaxis provide sterilizing immunity against RABV and genetically similar species, no such protection is conferred against the more genetically diverse lyssaviruses (19). Intrinsic innate immunity plays an important role in controlling the spread of numerous enveloped viruses; however, the influence of the IFITM ISGs on members of the *Lyssavirus* genus has not previously been evaluated. Although putative IFITM genes have been identified by database searching in many species (6, 9), no formal genome analysis or functional assessment of avian IFITM genes has been undertaken. Here we report the analysis of the IFITM locus, reaffirming the existence of chicken IFITM1 (chIFITM1) and providing the first functional characterization of chIFITM2 and chIFITM3, as well as demonstrating restriction of endosome-entering viruses by chIFITMs *in vitro*.

## MATERIALS AND METHODS

### Cell culture and generation of IFITM-expressing cell lines.

Human-derived A549 cells (CCL-185; ATCC) were grown in F-12 medium (Life Technologies) and human HEK293T (CRL-1573; ATCC) and chicken DF-1 (CRL-12203; ATCC) cells were grown in Dulbecco's modified Eagle's medium (DMEM) (Life Technologies); all media were supplemented with 10% (vol/vol) fetal bovine serum (FBS) (Biosera). Chicken and human IFITM gene sequences were synthesized (GeneArt; Life Technologies) as codon-optimized genes for expression in human cells, and chicken IFITMs were also synthesized for optimal expression in chicken cells. All IFITM genes were cloned into the BamHI and NotI sites of the lentivirus vector, pSIN-BNHA (20), and sequences confirmed by capillary sequencing (GATC Biotech). The gene cassette was cloned into pSIN-BNHA, to ensure that a C-terminal hemagglutinin (HA) tag followed the IFITM protein. Lentivirus vector stocks were made by a three-plasmid transfection of HEK293T cells, grown to confluence in a 10-cm dish. Briefly, 200 μl of Opti-MEM (Gibco) was mixed with 10 μl of Fugene-6 (Roche) before addition of 1 μg of a gag-pol-expressing vector (p8.91), 1 μg of a vesicular stomatitis virus glycoprotein (VSV-G)-expressing vector (pMDG), and 1.5 μg of vector expressing the transgene (pSIN-BNHA) and incubated for 15 min. The medium was removed from the cells and replaced with 8 ml of DMEM plus 10% fetal bovine serum (FBS), and the DNA mixture was added dropwise to the cells. After 24 h at 37°C and 5% CO_2_, the medium was removed and replaced with 8 ml DMEM plus 10% FBS and incubated for a further 24 h. Packaged lentivirus vector was harvested 48 and 72 h after transfection by collecting the supernatant and being filtered through a 0.45-μm-pore filter (Millex). The lentiviruses were used to transduce human A549 lung epithelial cells and produce a mixed population from which single cell clones were derived by limiting dilution. Expression of IFITMs was detected by HA flow cytometric analysis.

### Confocal microscopy.

Cells were seeded at 1 × 10^5^/well on coverslips in a 12-well plate 1 day prior to transfection with an IFITM-encoding plasmid (1 μg DNA with 3 μl of Fugene [Promega]). Cells were fixed with 100% methanol for 10 min followed by being blocked in 1% bovine serum albumin (BSA) for 30 min. The HA epitope was targeted by an anti-HA antibody conjugated to Alexa Fluor 550 (ab117513), and endosomes were visualized by a Lamp1 antibody with human (ab25630; Abcam) or chicken (LEP100 IgG; Developmental Studies Hybridoma Bank) specificity, followed by incubation with a secondary antibody conjugated to Alexa Fluor 488 (ab96871; Abcam).

### Flow cytometric analysis.

Transfected cells were harvested using 300 μl 0.25% trypsin-EDTA (Life Technologies), neutralized with 300 μl of cell culture medium plus 10% FBS, and pooled with the supernatant. The cells were spun at 2,000 × *g* for 5 min, and the pellet was resuspended in 100 μl PBS and transferred to a 96-well V-bottomed plate (Nunc). The plate was centrifuged, and the cells were fixed and permeabilized in 100 μl of Cytofix/Cytoperm buffer (Becton, Dickinson) and washed according to the manufacturer's guidelines. The cells were resuspended with the anti-HA antibody conjugated to fluorescein isothiocyanate (FITC) (A190-108F; Cambridge Bioscience) and incubated for 1 h at 4°C, followed by two rounds of washing. IAV replication was detected by antinucleoprotein (anti-NP) antibody (ab128193; Abcam) followed by incubation with anti-mouse Alexa Fluor 650 (ab96882; Abcam). Cells were resuspended in 300 μl of PBS before analysis by flow cytometry (FACSCalibur II; Becton, Dickinson).

### Infection of IFITM-expressing cell lines with pseudotyped viruses.

Chicken or human IFITM-expressing A549 cell lines were seeded at 3 × 10^3^ cells/well in 96-well plates 1 day prior to infection with either green fluorescent protein (GFP)-expressing pseudotyped lyssaviruses, RABV challenge virus standard 11 (CVS-11; GenBank accession no. EU352767) and Lagos bat virus (LBV) (LBV.NIG56-RV1; GenBank accession no. HM623779), luciferase-expressing pseudotyped influenza viruses (HA1 [GenBank accession no. AF117241], HA5 [GenBank accession no. EF541394], H7 [GenBank accession no. AJ491720], and H10 [GenBank accession no. CY014671]) or amphotrophic murine leukemia virus (MLV-A). GFP expression, as a measure of lentivirus infection, was determined by fluorescence microscopy at 48 h postinfection following fixation (20 min) with 4% (vol/vol) paraformaldehyde (USB) and permeabilization (10 min) using 0.3% Triton X–PBS. Cells were washed with 100 μl of PBS-Hoechst solution (Life Technologies) (200 ng/μl), and a plate seal was adhered. The fixed cells were analyzed to determine the proportion of cells expressing GFP (Cellomics ArrayScan VTI; Thermofisher), using the Target Activation bioapplication. Briefly, this method counts every cell on the plate by drawing a perimeter around each of the nuclei (detected by Hoechst) and calculates the percentage of these cells also expressing GFP. Luciferase activity, as a measure of lentivirus infection, was determined at 48 h postexposure using 50 μl Bright-Glo reagent (Promega). The cells were allowed to lyse for 2 min before the level of luciferase activity was measured using the FLUOstar Omega (BMG Labtech). GFP and luciferase levels are reported relative to infection of A549 cells in the absence of IFITM protein overexpression.

### siRNA knockdown studies.

DF-1 chicken cells were seeded at 5 × 10^4^ cells/well in a 24-well plate and transfected with a small interfering RNA (siRNA) against chIFITM3 (GCGAAGTACCTGAACATCACG) or a nonspecific siRNA (UUCUCCGAACGUGUCACGUGU), using Lipofectamine RNAiMAX (Life Technologies) 48 h prior to IFN stimulation. The cells were stimulated by addition of either 200 ng/ml of chicken IFN-γ (RP0115c; Kingfisher Biotech) or chicken IFN-α (PAP004; AbD Serotec) for a further 24 h or infected with IAV (A/WSN/1933 [WSN/33]) for 1 h at a multiplicity of infection (MOI) of 0.1. RNA was extracted according to the manufacturer's instructions (RNeasy minikit; Qiagen). Reverse transcription-PCR (RT-PCR) was performed (QuantiTect Multiplex RT-PCR kit; Qiagen) using probes and primers from ABI (chicken glyceraldehyde-3-phosphate dehydrogenase [GADPH], 4448489; and chicken_IFITM3, custom assay). Influenza virus infection was measured by flow cytometric analysis (see above) using an anti-NP antibody (ab20921; Abcam) to determine cell infection.

### Plaque assays.

Material to be assayed was serially diluted in serum-free DMEM and used to infect MDCK cells in 12-well plates. After 1 h of incubation, the inoculum was removed, and the cells were overlaid with DMEM containing 0.2% BSA (Sigma-Aldrich), 1.25% Avicel (FMC Biopolymer), and 1 μg trypsin ml^−1^ (21). After 2 days, the overlay was removed, and the cells were fixed with 4% formal saline–PBS solution for 20 min before being stained with 0.1% toluidine blue solution (Sigma-Aldrich) so that the number of PFU could be calculated.

### Expression of IFITM proteins in different chicken tissues.

Tissues were removed from 3-week-old specific pathogen-free (SPF) Rhode Island Red (RIR) chickens, specifically thymus, spleen, bursa of Fabricius, cecal tonsil, gastrointestinal tract, trachea, bone marrow, brain, muscle, heart, liver, kidney, lung, and skin. RNA was DNase treated, and reverse transcription was carried out (SuperScript III reverse transcriptase; Life Technologies). The cDNA from each tissue was amplified by PCR using the following primer sets: chIFITM1 (F′-AGCACACCAGCATCAACATGC, R′-CTACGAAGTCCTTGGCGATGA), chIFITM2 (F′-AGGTGAGCATCCCGCTGCAC, R′-ACCGCCGAGCACCTTCCAGG), chIFITM3 (F′-GGAGTCCCACCGTATGAAC, R′-GGCGTCTCCACCGTCACCA), and chicken_GAPDH (glyceraldehyde-3-phosphate dehydrogenase) (F′-ACTGTCAAGGCTGAGAACGG, R′-GCTGAGGGAGCTGAGATGA).

## RESULTS

### Identification of the chicken IFITM locus.

**Fig 1:**
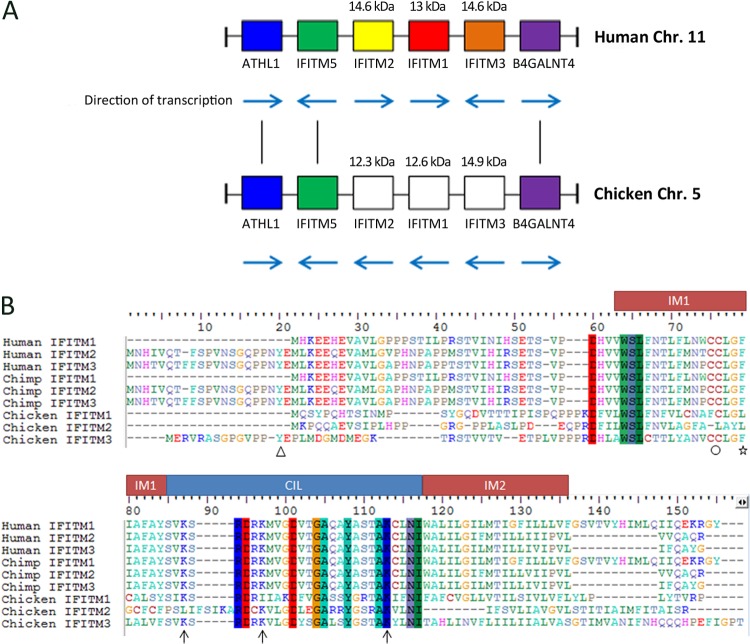
The chIFITM locus architecture and sequence. The *IFITM* gene cluster on Gallus gallus chromosome 5 is flanked by genes *ATHL1* and *B4GALNT4*. This region is syntenic with the *IFITM* gene cluster on human chromosome 11 (A). Note that the orientation change of *chIFITM2* and *chIFITM1* makes the assignment of orthology difficult; therefore, the chicken genes are named by gene order and conservation of specific functionally defined amino acid residues. The predicted mass is shown above the gene block. The colored columns in the sequence alignment (B) show residues that are shared between all nine *IFITM* sequences from humans, chimpanzees, and chickens. Significant residues have been highlighted with a symbol below the sequence: △, tyrosine; ○, double cysteine; star, phenylalanine important for multimerization; ↑, conserved ubiquitinated lysine. IM1, intramembrane 1; CIL, conserved intracellular loop; IM2, intramembrane 2.

The chicken genome (ENSEMBL browser, version 2.1) contains two putative IFITM genes on chromosome 5, the so-called *IFITM5* (ENSGALG00000004239; chromosome 5:1620304 to 1621805:1) and *IFITM10* (ENSGALG00000020497; chromosome 5:15244061 to 15249351:1). The putative *IFITM5* gene is located next to an uncharacterized gene (ENSGALG00000006478), with which it shares 30% amino acid identity. Immediately adjacent to this are three sequence gaps whose estimated sizes are 1 kb, 1 kb, and 400 bp in the ENSEMBL chicken genome build. Importantly, the putative IFITM genes in chicken are flanked by the telomeric β-1,4-*N*-acetyl-galactosaminyl transferase 4 (*B4GALNT4*) gene and the centromeric acid trehalase-like 1 (*ATHL1*) gene. The *B4GALNT4* and *ATHL1* genes flank the antiviral *IFITM 1*, *2*, -*3*, and -*5* gene block in mammalian genomes. Sequence similarity searches of the most recent build of the chicken genome (v4.0, NCBI) using TBLASTN analysis and the putative IFITM5 amino acid sequence, revealed several transcripts with high amino acid identity to IFITM5. Additionally, BLAST hits were also identified to putative genes LOC770612 and LOC422993, within the locus flanked by *B4GALNT4* and *ATHL1*. Between these putative genes, two BLAST hits span the exons of genes designated chicken *IFITM3-like* (NCBI, LOC422993; GenBank accession no. XM_420925.4) and *IFITM1-like* (NCBI LOC770612; GenBank accession no. XM_001233949.3). A third BLAST hit matches an uncurated gene, “gene 376074,” which is positioned between *IFITM3-like* and *IFITM5*. Further analysis of gene 376074 showed it shared amino acid sequence identity with both *IFITM3-like* and *IFITM1-like* genes. Sequence similarity searches of the NCBI chicken expressed sequence tag (EST) database suggests gene 376074 is expressed. All of the chIFITM paralogues, like mammalian IFITMs, are comprised of two exons, and the location of the intron-exon boundary is conserved across all of the chicken IFITM genes. Therefore, the chicken genome contains an intact IFITM locus with four putative IFITM genes flanked by the genes *B4GALNT4* and *ATHL1* (Fig. 1A).

### Annotation of the chicken IFITM genes.

Using genome synteny, we ascribe *chIFITM5* as orthologous to mammalian *IFITM5*, gene 376074 as orthologous to *IFITM2*, LOC422993 (*IFITM3-like*) as orthologous to *IFITM1*, and LOC770612 (*IFITM1-like*) as orthologous to *IFITM3*. Multiple amino acid sequence alignments between the three predicted antiviral chIFITM genes and direct orthologues in primate species suggest this assignment is plausible. A number of conserved IFITM family motifs are present in some of the chicken sequences (Fig. 1B), and although the chicken sequences differ significantly from the human and chimpanzee orthologues, many amino acids in the CIL domain are conserved. Multiple sequence alignments also reveal important amino acids in the chicken IFITMs that help to categorize each sequence as either IFITM1 or IFITM2/3. Tyr20 is conserved in all primate IFITM2 or -3 sequences and is also present in chicken “*IFITM1-like*” (NCBI) but none of the other *IFITM1* orthologues. This, as well as the longer N terminus, further supports our assessment of this gene as an *IFITM2* or -*3* gene, and by synteny, the gene is *IFITM3*. The alignment also reveals that other functionally significant amino acids are conserved in some of the chicken IFITM sequences, including the two cysteines (Cys75 and -76) in IM1 that are palmitoylation sites in other species (11) and are important for membrane positioning. Phe79, also in IM1, is conserved in chicken “*IFITM1-like*” (*chIFITM3*), which is believed to be important for mediation of a physical association between IFITM proteins (22). In light of syntenic analysis (and functional support shown later), we suggest the following revisions of the NCBI nomenclature: LOC770612 as *chIFITM3* (previously “*IFITM1-like*”), LOC422993 as *chIFITM1* (previously chicken “*IFITM3-like*”), and gene 376074 as *chIFITM2* (Fig. 1).

### Subcellular localization of IFITM proteins.

**Fig 2:**
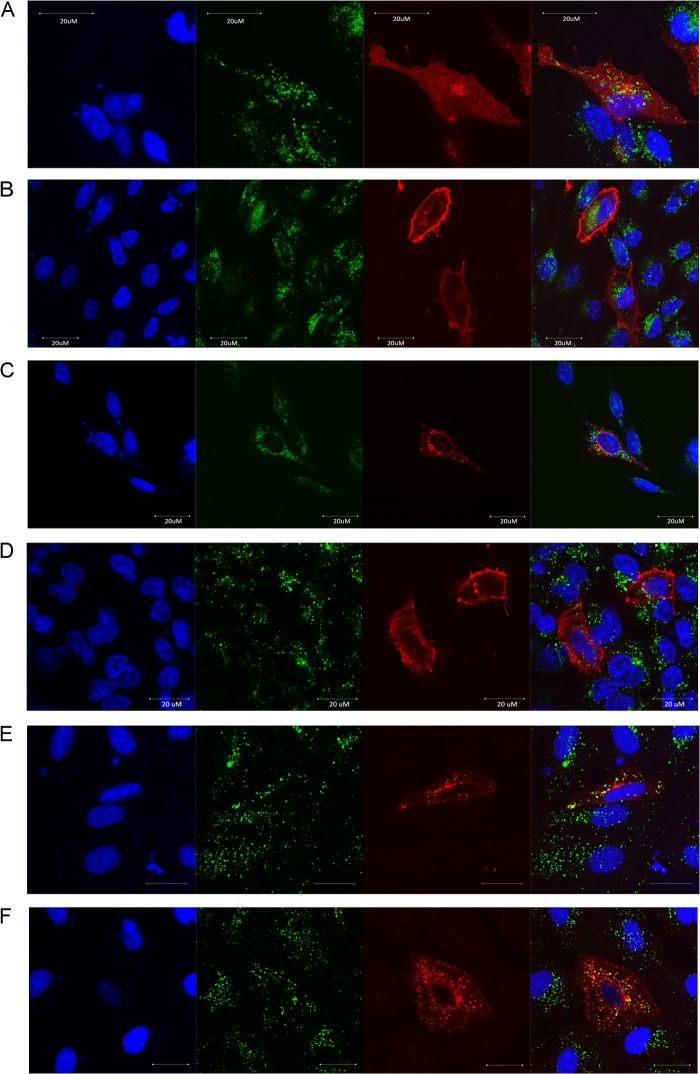
Cellular localization of overexpressed IFITM proteins. Confocal microscopy of DF-1 cells transfected with chIFITM proteins 1 to 3 (A, B, and C) and A549s transfected with huIFITM proteins 1 to 3 (D, E, and F) in the absence of infection. Panels show nuclei stained with DAPI (4′,6-diamidino-2-phenylindole) (blue), endosomes marked with an antibody against Lamp1 (green), IFITM protein marked by an antibody against the HA tag (red), and a merged image.

Human IFITM1, -2, and -3 localize distinctly in the cell, with IFITM1 being predominantly cell surface expressed and IFITM2 and -3 being predominantly intracellular, localizing with endosomes (Fig. 2D and E). The cellular localization of IFITM proteins can further delineate their orthologous relationships. We therefore synthesized codon-optimized, C-terminal HA-tagged chIFITM1, -2, and -3 and transiently transfected chicken DF-1 fibroblasts, comparing their cellular localization to the orthologous human IFITM (huIFITM) proteins expressed in human A549 cells. Confocal microscopy using an anti-HA antibody and an anti-LAMP1 antibody showed chIFITM1 was diffusely expressed throughout the cytoplasm, and chIFITM2 was present in the cytoplasm and the cell membrane, whereas chIFITM3 localized perinuclearly (Fig. 2A to C), consistent with huIFITM3. chIFITM3 therefore shares synteny, amino acid similarity, and subcellular localization with huIFITM3. In the case of the other two chIFITMs, their localization is a less clearly paired with the human IFITMs; thus, our nomenclature is founded on the gene order.

### Chicken IFITM proteins restrict diverse virus infection.

**Fig 3:**
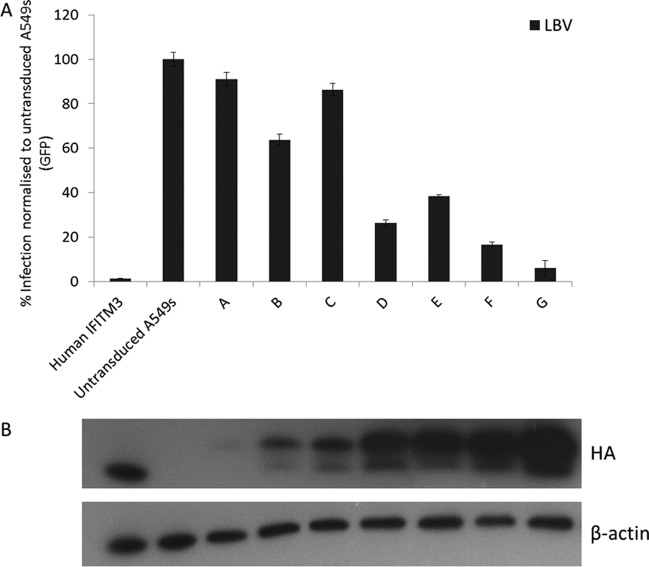
An increase in the expression of chIFITM3 is associated with a decrease in viral infection. A range of clonal A549 cell populations expressing increasing levels of chIFITM3 protein (bars A to G) were assessed by Western blotting of the HA tag (B). These cell lines were infected by a lentivirus pseudotyped with the Lagos bat virus (LBV) glycoprotein, and the replication was measured by GFP expression relative to that in untransduced A549s (A). Error bars show standard deviations of the means (*n* = 3).

**Fig 4:**
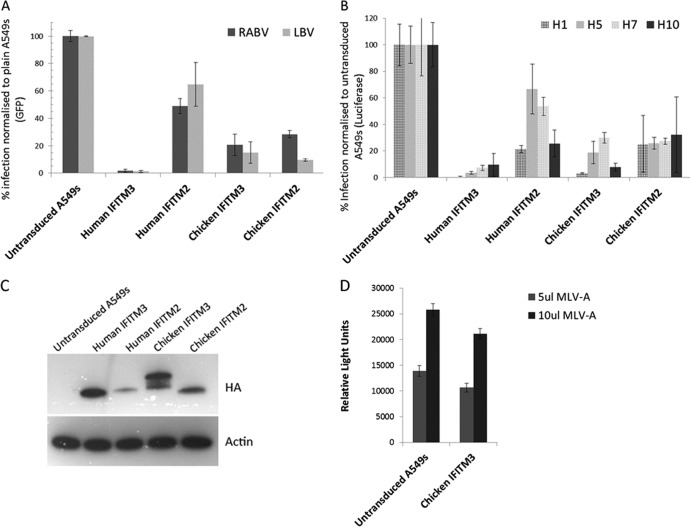
Human and chicken IFITM proteins restrict cell infection. Stable cell lines expressing human and chicken IFITM2 and -3 were infected by pseudotyped viruses with either lyssavirus glycoprotein envelopes (RABV [CVS-11]) and LBV (LBV.NIG56-RV1) (A) or IAV hemagglutinin envelopes (H1 [human], H5 [human], H7 [bird], or H10 [bird]) (B). The relative level of infection compared to untransduced A549 cells was measured by GFP expression or luciferase activity for the lyssavirus and IAV envelope pseudotypes, respectively. Error bars represent standard deviations across two biological replicates, each performed in triplicate. The expression levels of each cell line are shown by Western blotting (C) relative to endogenous B-actin. The stable cell line expressing chIFITM3 was infected with a pseudotyped virus expressing a luciferase reporter gene and the murine leukemia virus (MLV-A) envelope as a control (D).

We investigated if, despite considerable amino acid sequence divergence, chicken IFITMs could function as restriction factors. By expressing chIFITM3 at different levels in the cell, we show that there is a strong expression-level-dependent correlation between the level of chIFITM3 expression and the percentage of cells infected by a lentivirus vector pseudotyped with the lyssavirus envelope of LBV (Fig. 3). We then compared the level of antiviral restriction of chicken IFITM2 and -3 to that of their orthologous human proteins in A549 cells. Overexpression of *chIFITM3* resulted in 79.4% and 85% reductions in infection of A549s to lentivirus vectors pseudotyped with the lyssaviruses envelopes RABV and LBV, similar to the level of restriction by huIFITM3 to the same viruses (Fig. 4A), even though chickens are rarely infected by lyssaviruses (23). chIFITM2 also restricts lyssavirus LBV and RABV infection to a similar level as chIFITM3. A similar pattern of restriction is seen for lentiviruses pseudotyped with IAV H1, H5, H7, and H10 (Fig. 4B). huIFITM3 restricts viral infection of all influenza virus hemagglutinins, reducing infection by greater than 90%, and chIFITM3 restricts H1 and H10 pseudotypes as effectively, but restricts H5 and H7 less well. chIFITM2 and huIFITM2 restrict more moderately, as shown by others. Consistent with previous studies on huIFITM3 protein (1, 2), chIFITM3 failed to restrict MLV-A (Fig. 4D). Overall, although chIFITM3 and huIFITM3 only share 42% amino acid identity, the level of viral restriction of chIFITM3 is similar to that in huIFITM3. Data are not shown for chIFITM1, as a stably expressing cell line could not be made; this lack of stability at high expression levels is supported by Hach et al. (24), who show that overexpression of unpalmitoylated murine IFITM1 is difficult to achieve.

### Ablation of IFITM expression in chicken DF-1 cells increases infection.

**Fig 5:**
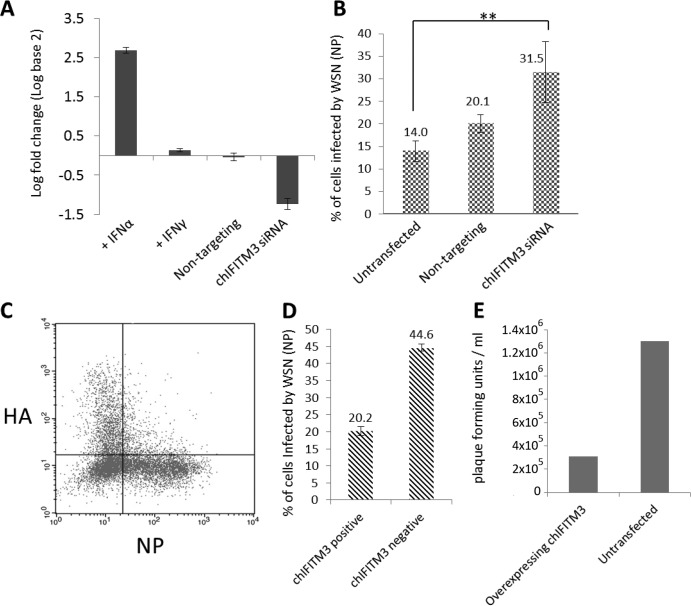
Chicken IFITM3 has an antiviral activity in DF-1 chicken cells. The expression level and log fold change of chIFITM3 were measured using quantitative RT-PCR after stimulation with IFN-α and IFN-γ or after preincubation with a nontargeting siRNA or one specific to chIFITM3 (A). The effect of knocking down endogenous chIFITM3 expression in DF-1 cells infected with influenza A virus (A/WSN/1933 [WSN/33]) was measured by flow cytometry using an antibody against nucleoprotein (NP) (B). *P* = 0.01, Student's *t* test. DF-1 cells transfected with chIFITM3-HA were infected by WSN. Expression of HA and NP was detected by flow cytometry (C and D), and viral titers were measured by PFU (E). Error bars represent standard deviations across each condition performed in triplicate.

We assessed the constitutive level of expression of *chIFITM3* in DF-1 cells, by quantitative RT-PCR with primers for *chIFITM3*. DF-1 cells abundantly express *chIFITM3* (threshold cycles [*C_T_*s] of 20 for IFITM3 and 22 for GAPDH). Despite being IFN inducible, addition of IFN-γ resulted in only a moderate induction, whereas addition of IFN-α caused a 2.67-log_2_ (6.4-fold) increase in *chIFITM3* expression (Fig. 5A). We assessed our ability to knock down *chIFITM3* expression in DF-1 cells using an siRNA designed to the *chIFITM3* transcript. Treatment with this siRNA on unstimulated DF-1 cells resulted in a 1.23-log_2_ (2.4-fold) reduction in the transcript level, with no change in *chIFITM3* transcript abundance with a nonspecific siRNA. Knockdown of endogenous *chIFITM3* resulted in a greater than 2-fold increase in infection of DF-1 cells by replication-competent influenza A virus (A/WSN/1933) (Fig. 5B), assayed by flow cytometric analysis of NP expression. Furthermore, overexpression of chIFITM3 in DF-1 cells reduced viral replication by an average of 55% (Fig. 5D), and plaque assays show that the viral load was reduced from 1.3 × 10^6^ PFU ml^−1^ to 3.1 × 10^5^ PFU ml^−1^ after chIFITM3 overexpression (Fig. 5E). Together, these results show chIFITM3 is able to restrict IAV entry into DF-1 cells.

### Differential expression of chIFITMs in chicken tissues.

**Fig 6:**
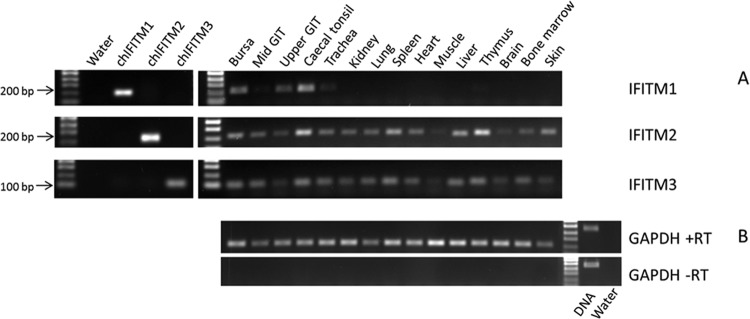
Differential expression of chIFITM transcripts in chicken tissues. Expression levels of IFITM1, -2, and -3 were determined by RT-PCR across a range of chicken tissues (A) and compared to the expression level of GAPDH (B). GAPDH PCR was performed without reverse transcriptase (−RT) to control for genomic DNA contamination.

We assessed the tissue-specific gene expression pattern in chickens using a panel of RNA extracted from tissues from 3-week-old Rhode Island Red (RIR) chickens focusing on thymus, spleen, bursa of Fabricius, cecal tonsil, trachea, gastrointestinal tract, bone marrow, brain, muscle, heart, liver, kidney, lung, and skin. Using primers specific to *chIFITM1*, -*2*, or -*3* (Fig. 6), expression of *chIFITM2* and -3 was detected in all tissues, although with lower levels of expression in the muscle and brain and higher levels in the cecal tonsils (Fig. 6). In contrast, expression of *chIFITM1* was more restricted and confined to the bursa of Fabricius, the gastrointestinal tract, and the cecal tonsil.

## DISCUSSION

To date, the antiviral activities of the IFITM2 and IFITM3 proteins have only been demonstrated in mammals, with a single report characterizing the function of chicken IFITM1 and IFITM5 (2). Computational analysis of vertebrate genomes suggests the IFITM gene family is present throughout vertebrates. However, this analysis and any phylogenetic reconstruction of gene history are complicated by the paralogous nature of the IFITM gene family, the presence of copy number variations, and the presence of numerous processed pseudogenes (6). Indeed, the identification of avian IFITMs as part of the dispanin protein family failed to identify chicken IFITMs in the antiviral IFITM1- to -3 subfamily defined as DSP2a to -c (25). Similarly, a more thorough analysis of vertebrate IFITMs, while identifying distantly related IFITMs in reptiles and birds, focused on eutherian clade 1 sequences for a detailed phylogenetic analysis (9). Hickford et al. (26) have undertaken a comprehensive analysis of IFITM genes across a broad range of chordates. The authors have shown that all of the species analyzed, including “lower” vertebrates, such as lampreys, possess at least one IFITM-like gene. Phylogenetic analysis of all of the IFITM paralogues they identified revealed that IFITM5 emerged in bony fish, while IFITM10 appears restricted to tetrapods. Here we have resolved the entire antiviral IFITM locus on chromosome 5 of the chicken genome, expanding the number of IFITM genes to 4 in this locus, and confirmed that the locus is flanked by the genes *ATHL1* and *B4GALNT4* (9). Crucially, we have shown that antiviral activity is conserved in chicken IFITMs. The low-level sequence identity and orientation change of chIFITM2 and chIFITM1 make the phylogenetic assignment of orthology problematic. Our revised nomenclature of the chicken IFITM locus is based on the syntenic gene order, as previously discussed, and functional data where possible. However, given chIFITM2 is localized to the plasma membrane, and the lack of an N-terminal extension (characteristic of huIFITM2/3), it is possible that it is analogous to huIFITM1. It is likely that similar extensive genetic and functional analyses will be essential to characterize the IFITM loci in other vertebrate species and define unambiguously IFITM1, -2, and -3 orthologues.

Using the chIFITM3 amino acid sequence, we also searched the duck genome (v1.0) and identified a scaffold (2943) containing two duck IFITM (duIFITM) orthologues. Sequence identity and conserved synteny with the chIFITM locus indicate they are IFITM5 and IFITM1. The two IFITM flanking genes, *B4GALNT4* and *ATHL1*, are also located on the scaffold in conserved positions. Although annotated gene structures are absent in the browser, IFITM cDNAs in other avian species align with the regions adjacent to both ends of the IFITM1 structure. This suggests the duck retains four IFITM genes at a conserved locus. We would expect the positions of duIFITM2 and duIFITM3 to be conserved with their orthologues in the chicken and other species. Following infection with two H5N1 strains of avian influenza virus (A/duck/Hubei/49/05 and A/goose/Hubei/65/05), levels of expression of duck IFITM3, -5, and -10 (measured by RNAseq) were increased to various degrees, reflecting a response befitting their expected function (27).

Control of animal pathogens, especially those with zoonotic potential, is a key component of ensuring human health and food security. RABV is responsible for approximately 70,000 human deaths each year (28), while other lyssaviruses have only been conclusively shown to cause a handful of fatalities (29), although this could be due to poor surveillance. Our results are the first to show diverse members of this genus of virus are sensitive to the inhibitory action of human IFITM proteins. Furthermore, although most warm-blooded animals are susceptible to RABV, domestic birds are rarely infected by lyssaviruses (23). Despite this, chIFITM2 and -3 were able to significantly reduce cell lyssavirus infection. Avian IAV infections, however, pose significant threats to human health, to the international poultry industry, and to small-scale poultry farmers (30). Our identification and functional characterization of the avian IFITM locus, together with knowledge that this gene family exists with copy number and allelic variants in other species (9, 15, 16), should provide a focus for identifying IFITM variants with enhanced antiviral activity for use in farm animal breeding strategies to improve animal infectious disease resistance. Specifically, we hypothesize that certain wild or outbred chicken IFITM allelic variants will confer enhanced levels of protection to pathogenic avian viruses that enter through acidic endosomes and that breeding for enhanced activity in IFITM variants will improve disease resistance in chickens. Similarly, should chicken IFITM proteins restrict IAV infection in chick embryos, the ablation of IFITM protein expression could improve vaccine production and boost yield.

We have shown that chIFITM proteins expressed in A549 cells are capable of restricting diverse viruses that enter cells through the acidic endosome pathway. Furthermore, we show that DF-1 chicken cells constitutively express *chIFITM3*, and this is able to restrict influenza virus infection *in vitro*. Despite sharing less than 50% amino acid identity, both *chIFITM3* and *huIFITM3* effectively restrict the entry of all lyssavirus and IAV envelope pseudotypes tested. Nevertheless, certain key amino acids in the N terminus, IM1, and CIL domain are conserved in chicken and human *IFITM3*, suggesting functional importance.

This work describes our elucidation of the IFITM locus in the chicken genome and provides the first functional characterization of chIFITM2 and chIFITM3. Despite this, many key questions remain; it is unclear how genes such as *IFITM3* in humans and chickens, separated by 310 million years of evolution (31) and sharing less than 50% amino acid identity, maintain a conserved cellular location and a strong antiviral activity against a diverse range of viruses. It is of equal importance to determine, given the level of antiviral activity and the proposed indirect mechanism of IFITM protein restriction (12, 13), how viruses overcome the restriction either within or between species. Investigation of appropriately defined IFITM loci from different host species where cross-species transfer of virus infection occurs may help explain barriers and vulnerabilities to infection by diverse viruses.
